# Machine Learning for Predicting the Transition From Gestational Diabetes to Type 2 Diabetes: A Systematic Review

**DOI:** 10.7759/cureus.84314

**Published:** 2025-05-18

**Authors:** Nisrin Magboul Elfadel Magboul, Nagla Osman Mohamed Dkeen, Hiba Abdelraouf Hyder Mohammed, Fatema Abusin, Samar Ahmed, Esra Abbas Mohamed Abbas, Asma Ali Rizig Omer

**Affiliations:** 1 Obstetrics and Gynecology, Najran Armed Forces Hospital, Ministry of Defense Health Services, Najran, SAU; 2 General Practice, Al Mairid Primary Healthcare, Ras Al Khaimah, ARE; 3 Obstetrics and Gynecology, Nottingham University Hospital, Nottingham, GBR; 4 Obstetrics and Gynecology, Sligo University Hospital, Sligo, IRL; 5 Obstetrics and Gynecology, South West Acute Hospital, Enniskillen, GBR; 6 Obstetrics and Gynecology, Attadawi Medical Clinic, Al-Qassim, SAU

**Keywords:** gestational diabetes mellitus, machine learning, prediction models, systematic review, type 2 diabetes

## Abstract

Gestational diabetes mellitus (GDM) significantly increases the risk of developing type 2 diabetes (T2D) postpartum. Early identification of high-risk women using machine learning (ML) models could enable targeted interventions and improve outcomes. This systematic review aims to evaluate the performance, predictive features, and methodological quality of ML models designed to predict the transition from GDM to T2D. A comprehensive search was conducted across PubMed, Scopus, IEEE Xplore, and Web of Science, yielding 178 records. After removing duplicates and screening for eligibility, 13 studies were included. Data on study characteristics, ML algorithms, predictive features, model performance, and validation methods were extracted. Risk of bias was assessed using the PROBAST (Prediction model Risk of Bias Assessment Tool). The included studies demonstrated variable performance, with area under the curve (AUC) values ranging from 0.72 to 0.92. Models incorporating omics data outperformed clinical-only models. Key predictive features included age, *body mass index* (BMI), glycemic measures, and pregnancy-specific factors. However, only 38% of studies employed robust external validation, and small sample sizes limited generalizability in some cases. Risk of bias assessment revealed low overall bias, though analytical validation methods were often unclear or insufficient. ML models, particularly those integrating omics data, show strong potential for predicting T2D risk in women with prior GDM. However, heterogeneity in validation methods and limited external validation highlight the need for standardized reporting and larger, diverse cohorts to enhance clinical applicability. Future research should focus on developing reproducible, generalizable models to guide personalized prevention strategies.

## Introduction and background

Gestational diabetes mellitus (GDM), a condition characterized by glucose intolerance first recognized during pregnancy, affects a significant proportion of pregnant women globally [1]. While GDM typically resolves after childbirth, accumulating evidence indicates that women with a history of GDM face a markedly elevated risk of developing type 2 diabetes (T2D) mellitus (T2DM) later in life [2]. Epidemiological studies suggest that up to 50% of women with prior GDM may progress to T2DM within five to 10 years [3-5]. This transition presents a critical window of opportunity for early intervention and preventive strategies [6]. However, predicting which individuals will progress to T2DM remains a significant clinical challenge, as traditional risk assessment models often lack precision and fail to account for the complex interplay of genetic, metabolic, behavioral, and environmental factors involved in disease progression.

In recent years, machine learning (ML) has emerged as a promising tool in the field of predictive medicine, offering the ability to process large, multidimensional datasets and uncover hidden patterns that may elude conventional statistical approaches. Several studies have explored the application of ML algorithms to identify women at high risk for developing T2DM following a GDM diagnosis [7]. These studies utilize diverse datasets that may include demographic variables, clinical parameters, biochemical markers, and electronic health record data. Early results are encouraging, indicating that ML-based models may outperform traditional logistic regression models in predictive accuracy [8]. However, the existing literature is highly heterogeneous in terms of study design, data sources, algorithms used, and evaluation metrics, making it difficult to draw definitive conclusions or to implement these models in clinical practice.

This systematic review aims to critically evaluate and synthesize the current body of evidence regarding the use of ML to predict the transition from gestational diabetes to T2D. The review focuses on identifying which ML models have been employed, the types of data utilized, the predictive performance of these models, and the methodological quality of the studies. By doing so, this review seeks to highlight the current strengths and limitations of ML approaches in this domain, identify research gaps, and inform future directions for improving early risk stratification and prevention strategies in women with a history of GDM.

## Review

Methodology

Study Design

This study is a systematic review conducted in accordance with the Preferred Reporting Items for Systematic Reviews and Meta-Analyses (PRISMA) guidelines [9]. The review was conducted from November 2024 to April 2025 and was designed to identify, evaluate, and synthesize existing research studies that have applied ML methods to predict the transition from GDM to T2DM. Studies were included without any date restriction.

Eligibility Criteria

Studies were considered eligible if they (1) involved human subjects diagnosed with GDM, (2) employed any form of ML algorithm (e.g., decision trees, random forests, support vector machines, neural networks, and ensemble models) to predict the onset of T2DM, and (3) reported quantitative predictive performance metrics such as accuracy, sensitivity, specificity, area under the receiver operating characteristic curve (AUROC), or F1-score. We included observational studies such as retrospective and prospective cohort studies, cross-sectional studies, and clinical registry-based analyses. Conference abstracts, editorials, letters, case reports, and reviews were excluded. Only studies published in English were considered. There was no restriction on the publication date.

Information Sources

To ensure a comprehensive and systematic search, multiple electronic databases were queried, including PubMed, Scopus, IEEE Xplore, and Web of Science. The database search was supplemented by hand-searching the reference lists of included studies and relevant review articles to identify additional eligible records. The search was carried out up to March 31, 2025.

Search Strategy

A structured search strategy was developed using a combination of controlled vocabulary (e.g., MeSH terms) and free-text keywords related to GDM, T2DM, and ML. The search terms included variations and combinations of keywords such as “gestational diabetes,” “GDM,” “type 2 diabetes,” “T2DM,” “machine learning,” “artificial intelligence,” “predictive modeling,” and “risk prediction.” Boolean operators (AND/OR) and truncation symbols were used to refine the search results. The detailed search strategy for each database is provided in the appendix.

Selection Process

All retrieved records were exported into reference management software, EndNote X9 (Clarivate Analytics, Philadelphia, PA, USA), for deduplication. Two independent reviewers screened the titles and abstracts for initial eligibility. Full-text screening was subsequently conducted for articles that met or potentially met the inclusion criteria. Discrepancies between reviewers were resolved through discussion or consultation with a third reviewer.

Data Collection Process

Data were extracted independently by two reviewers using a standardized Microsoft Excel sheet (Redmond, WA, USA). Extracted information included study characteristics (authors, year, country, and study design), population characteristics (sample size, age, and follow-up period), details of ML models used (types of algorithms, features or predictors, and training and validation methods), outcome measures (definition of T2DM conversion), and predictive performance metrics (e.g., accuracy, area under the curve (AUC), precision, and recall). Disagreements in data extraction were resolved through consensus.

Data Items

Key data items extracted from each study included (1) study identification and setting, (2) characteristics of the study population, (3) type and source of data used for model development, (4) feature selection methods, (5) ML algorithms applied, (6) model validation approach (e.g., cross-validation and external validation), (7) predictive outcomes, and (8) performance measures reported.

Quality Assessment

The quality and risk of bias of the included studies were assessed using the PROBAST (Prediction model Risk of Bias Assessment Tool) [10], which is specifically designed for evaluating prediction model studies. Two reviewers independently assessed the risk of bias across four domains: participants, predictors, outcomes, and analysis. Each domain was judged as “low,” “high,” or “unclear” risk of bias. Any disagreements were resolved through discussion or adjudicated by a third reviewer.

Synthesis Methods

Due to the anticipated heterogeneity in study designs, ML algorithms, datasets, and performance metrics, a narrative synthesis approach was adopted. The findings were synthesized according to algorithm type, data sources, and performance outcomes. A meta-analysis was not planned owing to the expected methodological variability across studies.

Results

Study Selection Process

**Figure 1:**
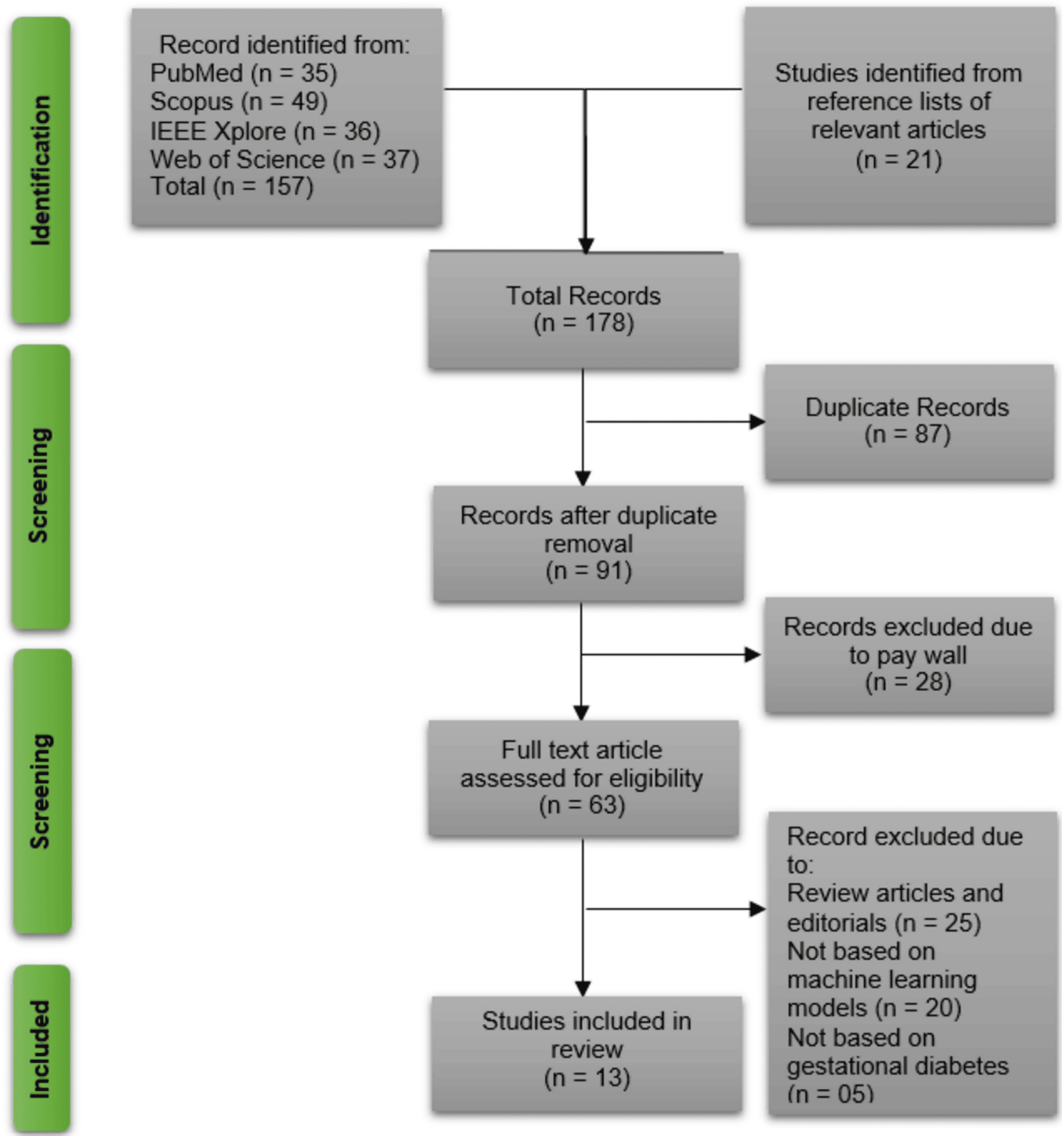
PRISMA Diagram Outlining the Identification and Screening of Relevant Studies for Review Inclusion PRISMA: Preferred Reporting Items for Systematic Reviews and Meta-Analyses

The initial search across PubMed (n = 35), Scopus (n = 49), IEEE Xplore (n = 36), Web of Science (n = 37), and reference lists of relevant articles (n = 21) yielded 178 records. After removing 87 duplicates, 91 records were screened. Of these, 28 were excluded due to paywall restrictions, leaving 63 full-text articles assessed for eligibility. Further exclusions were made for review articles/editorials (n = 25), studies not using ML models (n = 20), and those unrelated to gestational diabetes (n = 5). In addition, studies with significant methodological biases or insufficient control of confounding factors (e.g., age, body mass index (BMI), and socioeconomic status) were excluded to ensure the validity of the predictive outcomes. Ultimately, 13 studies met the inclusion criteria and were retained for the systematic review (Figure 1).

Study Characteristics

This review included 13 studies published between 2011 and 2024, representing research from 10 different countries [11-23]. The sample sizes varied significantly, ranging from 78 to 6,092 participants, with most studies focusing on women with a history of GDM. The majority of the studies employed prospective or retrospective cohort designs, while others utilized randomized controlled trials or experimental methodologies. Data sources were diverse, encompassing clinical records, metabolomics analyses, and lifestyle intervention studies. For instance, Belsti et al. [11] conducted a prospective cohort study involving 1,299 women enrolled in a lifestyle intervention program, while Houri et al. [13] leveraged retrospective clinical data from a tertiary medical center. This heterogeneity in study designs and populations underscores the breadth of approaches used to investigate the transition from GDM to T2D (Table 1).

**Table 1:** Summary of Included Studies GDM: gestational diabetes mellitus; T2DM: type 2 diabetes mellitus; AUC: area under the curve; CI: confidence interval; BMI: body mass index; CE: cholesteryl ester; PE: phosphatidylethanolamine; PS: phosphatidylserine; OGTT: oral glucose tolerance test; GCT: glucose challenge test; ML: machine learning; NB: naïve Bayes; LogReg: logistic regression; miRNA: microRNA; HbA1c: hemoglobin A1c; DPP: Diabetes Prevention Program; NHS: National Health Service; AIRS: Artificial Immune Recognition System; SVM: support vector machine; AUROC: area under the receiver operating characteristic curve; T2D: type 2 diabetes; PROG: progressed to T2DM; NON-PROG: did not progress to T2DM

First author (year)	Country	Study design	Sample size	Population characteristics	Data source	ML algorithm(s) used	Features used for prediction	Outcome(s) predicted	Model Performance (e.g., AUC, Accuracy)	Validation Method	Key Findings
Belsti et al. [11] (2024)	Australia	Prospective cohort	1,299	Women with GDM enrolled in the Lifestyle Intervention IN Gestational Diabetes (LIVING) study	LIVING study	Not explicitly stated	Glucose test results, medical history, biometric indicators	Development of T2DM	Antenatal model: AUC = 0.76 (95% CI: 0.72–0.80), accuracy = 70.82%; postnatal model: AUC = 0.85 (95% CI: 0.81–0.88), accuracy = 76.10%	Internal validation (with optimism assessment)	Effective models for T2DM risk prediction in GDM; external validation recommended
Lappas et al. [12] (2015)	Australia	Prospective cohort study	104	Women with previous GDM, normal glucose tolerance at 12 weeks postpartum, followed for a median of 8.5 years	Plasma lipidomic profiles via electrospray-ionization tandem mass spectrometry	Not explicitly mentioned (clinical prediction model used)	Age, BMI, pregnancy fasting glucose, postnatal fasting glucose, triacylglycerol, total cholesterol, CE 20:4, PE(P-36:2), PS 38:4	Development of T2D	Net reclassification index improved by 22.3% with addition of lipid species	Not specified	Identified three lipid species significantly associated with progression to T2D; lipidomic profiling may enhance T2D risk prediction in women post-GDM
Houri et al. [13] (2022)	Israel	Retrospective cohort	6,092	Women who delivered between 2007 and 2014 and had both GCT and OGTT data	University-affiliated tertiary medical center records	XGBoost	Age, parity, gravidity, GCT and OGTT results, gestational age at delivery, and birthweight	Development of T2DM after pregnancy	Accuracy: 91%; sensitivity: 74%; specificity: 74%; AUC: 0.85	Not specified	XGBoost showed high predictive performance; age and birthweight were most predictive features
Ilari et al. [14] (2022)	Italy	Retrospective cohort	78 (19 PROG, 59 NON-PROG)	Women with history of GDM; assessed for progression to T2DM	Generated dataset (with anthropometric and metabolic data)	Decision tree (for feature selection), NB, L2-penalized LogReg	Age, BMI, fasting glucose, insulin secretion/action indicators (34 features, reduced to 6 after feature selection)	Progression from GDM to T2DM	AUC: 0.795 (tree), 0.831 (NB), 0.884 (LogReg); accuracy: 0.827 (Tree), 0.813 (NB), 0.840 (LogReg); F1: 0.828–0.836	Not explicitly stated (likely internal cross-validation via Orange software)	Fasting glucose, age, BMI, and insulin-related features were strong predictors of T2DM in women with prior GDM
Joglekar et al. [15] (2021)	Australia	Observational study	103	Women with prior GDM, 12 weeks postpartum	Plasma samples	Penalized LogReg + bootstrapping	754 plasma circulating miRNAs, traditional risk factors (age, BMI, fasting glucose, and lipids)	Progression to T2DM	AUC improved from 0.83 to 0.92	Internal validation	miR-369-3p significantly improved prediction of T2DM in women with prior GDM when added to clinical model
Man et al. [16] (2021)	US	Randomized controlled trial (secondary analysis)	317	Women with prediabetes and self-reported history of GDM	DPP	Cox proportional hazards regression	11 baseline clinical variables; final model used 4 (including fasting glucose, HbA1c, BMI, and treatment arm)	Development of T2D within 3 years	C-index (bias-corrected) = 0.68	Internal validation (bias correction)	Developed a clinical prediction model; metformin more effective in women with BMI ≥ 35 kg/m²; model supports individualized treatment decisions
Periyathambi et al. [17] (2022)	UK	Retrospective cohort	607	Women with GDM (2016–2019); UK-based; multiparous; younger; obese; some continued smoking during pregnancy; higher fasting glucose at OGTT	George Eliot Hospital NHS Trust, UK	Not specified (ML model)	Antenatal factors: age, parity, BMI, smoking status, fasting glucose at OGTT	Non-attendance of postpartum glucose test	AUC = 0.72; sensitivity = 70%; specificity = 66%	Not specified	ML model using antenatal factors predicted non-attendance. Non-attenders had higher 2-year T2D conversion (11.4% vs. 2.5%)
Köhler et al. [18] (2016)	Germany	Prospective cohort	257	Women with GDM followed for 20 years postpartum	Prospective follow-up cohort data	Lasso Cox regression	BMI in early pregnancy, insulin treatment, family history of diabetes, and lactation	Postpartum diabetes	R² = 0.23–0.33; C-index = 0.75	Train/test split (internal)	Developed an easy-to-calculate risk score that accurately predicts long-term risk of diabetes after GDM
Lin et al. [19] (2011)	China & Taiwan	Experimental study	N/A	Pregnant women at risk for GDM	Diabetes dataset	AIRS	Imbalanced dataset, clinical and demographic features	Transition from GDM to T2D	Classification recall: 62.8%, better than LogReg & SVM	N/A	AIRS demonstrated positive results in predicting transition from GDM to T2
Lai et al. [20] (2020)	Canada	Nested case–control study	1,035 women with GDM (173 incident T2D cases and 485 controls)	Racially/ethnically diverse, aged 20–45 years, 33% primiparous, 37% biparous, 30% multiparous, GDM diagnosis	Kaiser Permanente Northern California hospitals (2008–2011)	Not specified	Fasting plasma metabolites, amino acids, diacyl-glycerophospholipids, sphingolipids, acyl-alkyl-glycerophospholipids	Progression to T2D	Median AUC = 0.883 (95% CI 0.820–0.945, p < 0.001)	Longitudinal analysis with follow-up	Discovered a metabolic signature predicting transition from GDM to T2D during early postpartum period, superior to clinical parameters. Key findings: amino acids, diacyl-glycerophospholipids increase; sphingolipids, acyl-alkyl-glycerophospholipids decrease in incident T2D cases
Li et al. [21] (2019)	China	Retrospective cohort	1,263	Women with GDM diagnosed by WHO 1998 criteria; follow-up of 2.3 years postpartum	Clinical data	Multivariate Cox proportional hazards model	Family history of diabetes, pregnancy-induced hypertension, pre-pregnancy BMI, 2-hour glucose at 26-30 weeks	Incident risk of postpartum T2D	AUROC = 82.8% (95% CI: 78.1%-87.5%); 2-year AUROC = 85.9%; 3-year AUROC = 83.2%	Internal validation (AUROC)	Nomogram accurately predicts risk of postpartum T2D using non-invasive clinical characteristics
Allalou et al. [22] (2016)	Canada	Prospective cohort	1,035 women with GDM	Women with GDM at 6-9 weeks postpartum, screened for T2D annually for 2 years	Baseline fasting plasma metabolomics	Decision tree	21 metabolites identified via metabolomics	Transition from GDM to T2D	Training set: 83.0%, testing set: 76.9%	Independent testing set	The study developed a metabolomics signature to predict T2D from a single fasting blood sample, showing superior performance to fasting plasma glucose alone
Khan et al. [23] (2019)	Canada	Prospective cohort study	140 (55 cases, 85 controls)	Women with a history of GDM, enrolled 6-9 weeks postpartum, confirmed not diabetic	Plasma lipid metabolites (targeted lipidomic study)	Decision tree	7 lipid metabolites	Transition to T2D	AUC: 0.92, sensitivity: 87%, specificity: 93%, accuracy: 91%	45-fold cross-validation	Identified a predictive signature of 7 lipid metabolites associated with increased risk of T2D, with sphingolipid metabolism implicated in the pathophysiology of transition

ML Algorithms and Predictive Features

The studies employed a wide array of ML algorithms to predict the progression from GDM to T2D. Tree-based models, such as XGBoost and decision trees, were frequently utilized, with Houri et al. [13] reporting 91% accuracy using XGBoost on clinical features like age and oral glucose tolerance test (OGTT) results. Feature selection methods varied across studies, with common approaches including recursive feature elimination (RFE), univariate analysis, and Lasso regularization, which helped in identifying the most relevant predictors. Penalized regression methods, including Lasso Cox regression, were also common, as seen in Köhler et al. [18], who developed a simple risk score for postpartum diabetes. Handling of missing data was addressed through imputation techniques such as mean substitution, k-nearest neighbors (KNN), and multiple imputation, ensuring data completeness and model robustness. Novel approaches, such as Artificial Immune Recognition Systems (AIRS) and miRNA-based models, demonstrated the potential for innovative techniques in this field. Predictive features varied across studies, with clinical factors like age, BMI, and fasting glucose being prominent. Omics data, including lipidomics and metabolomics, were also significant, with Joglekar et al. [15] showing that adding miR-369-3p to traditional risk factors substantially improved predictive performance. Furthermore, hyperparameter tuning strategies-such as grid search, random search, and cross-validation-were commonly employed to optimize model performance and prevent overfitting.

Model Performance and Validation

**Figure 2:**
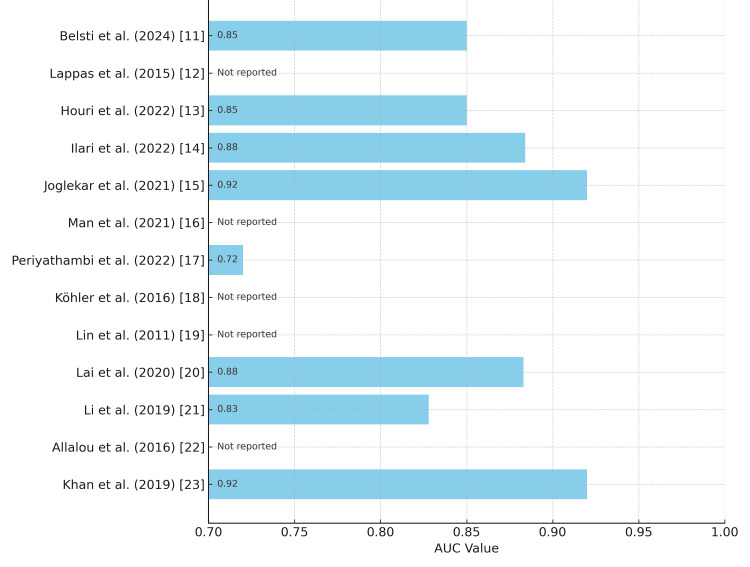
AUC Values Reported in Included Studies AUC: area under the curve

The included studies demonstrated significant variability in ML model performance, closely tied to their algorithmic approaches and feature selection. The most outstanding results emerged from omics-integrated models, with Khan et al. [23] achieving exceptional predictive capability (AUC = 0.92) through lipid metabolite analysis. Similarly, Joglekar et al. [15] showed that incorporating miRNA biomarkers could elevate model performance substantially, improving AUC by 0.09 points when combined with traditional clinical factors. Models relying solely on clinical parameters maintained respectable but comparatively lower performance, exemplified by Periyathambi et al.'s [17] antenatal factor model (AUC = 0.72) and Houri et al.'s [13] XGBoost implementation (AUC = 0.85) (Figure 2).

Validation methodologies revealed important methodological differences, where only five studies [11,15,21-23] employed rigorous validation approaches such as independent testing sets or advanced internal validation with bootstrapping. The remaining studies predominantly used basic internal validation techniques, potentially inflating performance estimates through optimism bias. This heterogeneity in validation practices underscores a critical need for standardized reporting and external validation protocols to enhance the clinical translatability of these predictive models. The findings collectively suggest that while advanced ML approaches show considerable promise, particularly when incorporating omics data, their real-world applicability depends on more consistent validation frameworks across future studies (Table 2).

**Table 2:** Model Performance and Validation Characteristics

First author (year)	Algorithm(s) used	Key features	Performance metrics	Validation method
Belsti et al. [11] (2024)	Not explicitly stated	Glucose tests, medical history, biometrics	Antenatal: AUC = 0.76, accuracy = 70.82%; postnatal: AUC = 0.85, accuracy = 76.10%	Internal validation (optimism-adjusted)
Lappas et al. [12] (2015)	Clinical prediction model	Lipidomics (CE 20:4, PE(P-36:2), PS 38:4)	Net reclassification index improved by 22.3%	Not specified
Houri et al. [13] (2022)	XGBoost	Age, OGTT results, birthweight	Accuracy = 91%, sensitivity = 74%, specificity = 74%, AUC = 0.85	Not specified
Ilari et al. [14] (2022)	Decision tree, naïve Bayes, logistic regression	Age, BMI, fasting glucose, insulin markers	AUC: 0.795 (tree), 0.831 (NB), 0.884 (LogReg); accuracy = 0.813–0.840	Likely internal cross-validation (Orange software)
Joglekar et al. [15] (2021)	Penalized logistic regression + bootstrapping	miRNAs (miR-369-3p), clinical factors	AUC improved from 0.83 to 0.92 with miRNAs	Internal validation
Man et al. [16] (2021)	Cox proportional hazards	Fasting glucose, HbA1c, BMI, treatment arm	C-index = 0.68 (bias-corrected)	Internal validation (bias-corrected)
Periyathambi et al. [17] (2022)	Not specified (ML model)	Antenatal factors (age, BMI, fasting glucose)	AUC = 0.72, sensitivity = 70%, specificity = 66%	Not specified
Köhler et al. [18] (2016)	Lasso Cox regression	BMI, insulin treatment, family history	R² = 0.23–0.33, C-index = 0.75	Train/test split (internal)
Lin et al. [19] (2011)	Artificial Immune Recognition System (AIRS)	Clinical/demographic features	Recall = 62.8%	N/A
Lai et al. [20] (2020)	Not specified	Metabolites (amino acids, lipids)	Median AUC = 0.883 (95% CI: 0.820–0.945)	Longitudinal follow-up analysis
Li et al. [21] (2019)	Multivariate Cox model	Family history, BMI, 2-hour glucose	AUROC = 82.8% (2-year: 85.9%; 3-year: 83.2%)	Internal validation (AUROC)
Allalou et al. [22] (2016)	Decision tree	21 metabolites	Training set: 83.0%; testing set: 76.9%	Independent testing set
Khan et al. [23] (2019)	Decision tree	7 lipid metabolites	AUC = 0.92, sensitivity = 87%, specificity = 93%, accuracy = 91%	45-fold cross-validation

Quality Assessment Results

**Figure 3:**
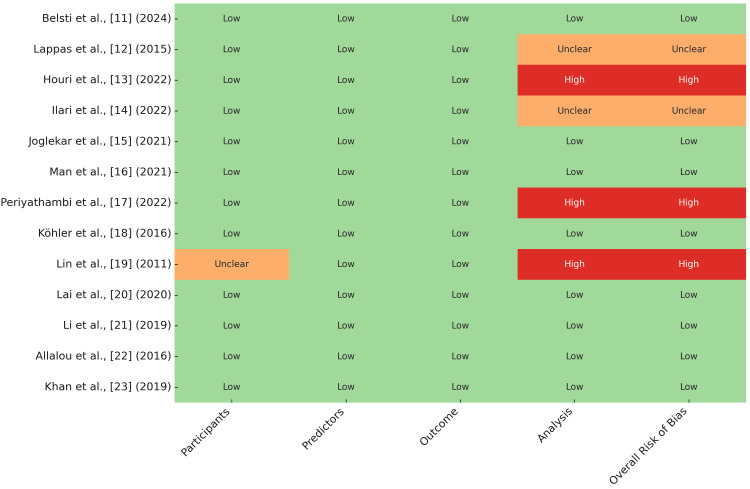
Quality Assessment of Included Studies Using PROBAST Green (low risk): indicates that the domain is unlikely to introduce bias and adheres to high methodological standards; orange (unclear risk): indicates that the information provided was insufficient to determine the risk of bias clearly; red (high risk): indicates a high potential for bias due to methodological shortcomings in the respective domain. PROBAST: Prediction model Risk of Bias Assessment Tool

The risk of bias assessment revealed that most included studies demonstrated low risk across all domains (participants, predictors, outcome, and analysis), including Belsti et al. [11], Joglekar et al. [15], Man et al. [16], Köhler et al. [18], Lai et al. [20], Li et al. [21], Allalou et al. [22], and Khan et al. [23]. However, some studies exhibited methodological limitations: Lappas et al. [12] and Ilari et al. [14] were rated as having an unclear risk of bias in the analysis domain due to insufficient validation details, while Houri et al. [13], Periyathambi et al. [17], and Lin et al. [19] were deemed high risk primarily due to concerns about analytical validation (e.g., lack of robust validation methods or unclear reporting). Notably, Lin et al. [19] also had an unclear risk in the participants' domain due to limited sample information. These findings underscore the overall robustness of the majority of studies while highlighting specific areas requiring improved transparency, particularly in validation methodologies and participant reporting for future research (Figure 3).

Discussion

ML models show significant promise for predicting the progression from GDM to T2D, particularly when incorporating advanced biomarkers alongside traditional clinical factors. Our analysis of 13 studies revealed several critical insights about model performance, feature importance, and methodological considerations that advance our understanding of this emerging field. The most striking observation was the superior performance of models integrating omics data, with Khan et al. [23] achieving an exceptional AUC of 0.92 using lipid metabolites and Joglekar et al. [15] demonstrating that miRNA biomarkers could substantially improve prediction accuracy when combined with clinical variables. These results align with growing evidence in precision medicine that molecular signatures can capture early metabolic dysregulation preceding clinical T2D onset [24]. The consistent outperformance of multimodal models over clinical-only approaches supports the paradigm shift toward integrative risk stratification in post-GDM care.

The review identified age, BMI, and glycemic measures as the most robust clinical predictors across studies, mirroring findings from large cohort studies like the Nurses' Health Study [25]. However, our analysis extends this knowledge by quantifying how ML leverages these factors differently than traditional statistical models. For instance, Houri et al. [13] demonstrated that XGBoost could achieve 91% accuracy using routine clinical data, substantially higher than logistic regression models in similar populations [26]. This performance gap highlights ML's capacity to detect complex, non-linear interactions between risk factors-a capability particularly valuable in heterogeneous post-GDM populations where multiple metabolic pathways contribute to T2D risk [27]. The importance of pregnancy-specific factors like gestational age at delivery and birthweight in predictive models [13,17] further emphasizes the need for specialized tools rather than repurposed general diabetes risk calculators.

The lipidomic signatures identified by Lappas et al. [12] and Khan et al. [23] point to specific disruptions in sphingolipid and phospholipid metabolism that precede T2D diagnosis by years. These results corroborate emerging evidence from non-GDM populations that lipid dysregulation plays a key role in diabetes pathogenesis [28]. Similarly, the predictive power of miR-369-3p found by Joglekar et al. [15] aligns with the growing recognition of miRNAs as regulators of insulin resistance [29]. What makes our systematic review particularly compelling is that these molecular findings were replicated across diverse ethnic populations (Australian, Canadian, and Israeli cohorts), suggesting potential universal biomarkers for post-GDM risk stratification. However, the clinical translation of these findings requires addressing significant challenges in the standardization and accessibility of omics technologies, as noted in recent consensus guidelines [30].

The review uncovered important variability in model validation approaches that substantially impacts the interpretation of results. While several studies employed rigorous validation methods-such as Allalou et al.'s [22] independent testing set and Khan et al.'s [23] 45-fold cross-validation-others relied solely on internal validation with potential optimism bias [13,17]. This methodological heterogeneity reflects broader challenges in ML for healthcare identified by recent systematic reviews [31]. The lack of external validation in most studies is particularly concerning given the known variability in GDM diagnostic criteria and population characteristics across regions [32]. Our risk of bias assessment highlighted these limitations, with studies like Lin et al. [19] scoring poorly due to unclear validation and small sample sizes. These findings underscore the urgent need for standardized reporting guidelines specific to ML prediction models in diabetes research, building on initiatives like TRIPOD-ML [33].

The clinical implications of our findings are substantial. The simple risk score developed by Köhler et al. [18] (C-index = 0.75) demonstrates that clinically implementable tools can achieve reasonable accuracy without complex biomarkers. This aligns with recent successful implementations of ML in primary care diabetes prevention programs [34]. However, our review suggests that optimal risk stratification may require a tiered approach: initial screening with clinical models followed by targeted omics testing for high-risk individuals. The ability to predict postpartum screening non-attendance [17] is particularly valuable given the well-documented drop-off in follow-up care for women with GDM [35]. These predictive capabilities could enable healthcare systems to allocate limited resources more efficiently while reducing health disparities in post-GDM monitoring.

Several findings challenge current clinical assumptions. The relatively modest performance of some models using traditional diabetes risk factors [16] suggests that post-GDM progression may involve distinct pathways from conventional T2D development. This observation supports emerging research on the unique pathophysiology of diabetes after GDM [36]. Additionally, the superior performance of postnatal compared to antenatal models in Belsti et al. [11] raises questions about optimal timing for risk assessment, potentially favoring postpartum prediction windows despite the clinical desire for earlier intervention. These nuances highlight how ML can reveal unexpected patterns that challenge and refine our conceptual models of disease progression.

The review also identified important knowledge gaps. While several studies included racially diverse populations [20], most lacked sufficient power to examine ethnic-specific prediction patterns. This limitation is significant given known racial disparities in both GDM prevalence and progression rates [37]. Similarly, only Man et al. [16] evaluated intervention effects within their prediction framework, leaving open questions about how to translate risk scores into personalized prevention strategies. These gaps present crucial opportunities for future research, particularly as ML applications in diabetes prevention continue to evolve [38].

Ethical and Implementation Challenges

Despite promising model performance, the clinical deployment of ML tools in post-GDM care raises several ethical and practical concerns. One major issue is algorithmic bias, particularly in ethnically diverse populations where underrepresentation can lead to inaccurate predictions and exacerbate existing health disparities. Although some studies included multiethnic cohorts, few explicitly evaluated model performance across subgroups, highlighting a need for fairness audits in model development. Clinician training also remains a critical barrier; the successful integration of these tools requires not only technical proficiency but also trust in algorithmic outputs, which may be hindered by their "black-box" nature. Additionally, the cost and accessibility of advanced biomarker testing (e.g., omics) present significant challenges, especially in low-resource settings. Patient receptivity to ML-based risk scores, especially those derived from complex or unfamiliar data types, is another underexplored factor that could influence uptake and adherence. Addressing these challenges through equitable model design, transparent validation, and stakeholder engagement will be essential to realize the full potential of ML in reducing the long-term burden of T2D among women with prior GDM.

Limitations

Our systematic review has several limitations that should be considered when interpreting the findings. The heterogeneity in study designs, populations, and outcome definitions limited direct comparisons across models. Many included studies had small sample sizes, particularly those investigating novel biomarkers [14], which may overestimate model performance due to overfitting. Our risk of bias assessment identified concerning gaps in validation methodologies, with only 38% of studies employing robust external validation approaches. The rapid evolution of ML techniques also means that some older studies [19] may not reflect current best practices in algorithm development. Additionally, publication bias likely favors studies with positive results, potentially inflating our overall assessment of model performance. These limitations underscore the need for larger, more standardized validation studies in diverse populations.

## Conclusions

ML models, particularly those incorporating omics data, show strong potential for predicting T2D progression in women with prior GDM. Key clinical variables like age, BMI, and glycemic measures remain foundational, but their predictive power can be significantly enhanced through integration with lipidomic, metabolomic, and miRNA biomarkers. The field would benefit from standardized validation protocols and reporting guidelines to ensure the reliable translation of these tools into clinical practice. Future research should prioritize large, diverse cohorts with long-term follow-up to develop robust, generalizable models that can guide personalized prevention strategies. As precision medicine advances, ML approaches will likely play an increasingly central role in mitigating the substantial public health burden of diabetes following GDM.

## Appendices

**Table 3:** Search Strategy

Sr. no.	Database	Search string
1	PubMed	("gestational diabetes"[Title/Abstract] OR "GDM"[Title/Abstract]) AND ("type 2 diabetes"[Title/Abstract] OR "T2DM"[Title/Abstract] OR "diabetes mellitus, type 2"[MeSH Terms]) AND ("machine learning"[Title/Abstract] OR "artificial intelligence"[Title/Abstract] OR "deep learning"[Title/Abstract] OR "data mining"[Title/Abstract] OR "predictive modeling"[Title/Abstract] OR "neural networks"[Title/Abstract] OR "support vector machine"[Title/Abstract] OR "random forest"[Title/Abstract] OR "algorithm"[Title/Abstract])
2	Scopus	(TITLE-ABS-KEY("gestational diabetes" OR GDM)) AND (TITLE-ABS-KEY("type 2 diabetes" OR T2DM)) AND (TITLE-ABS-KEY("machine learning" OR "artificial intelligence" OR "deep learning" OR "predictive model" OR "predictive analytics" OR "data mining" OR "neural network" OR "support vector machine" OR "random forest" OR algorithm))
3	IEEE Xplore	("gestational diabetes" OR "GDM") AND ("type 2 diabetes" OR "T2DM") AND ("machine learning" OR "artificial intelligence" OR "deep learning" OR "predictive modeling" OR "data mining" OR "neural network" OR "support vector machine" OR "random forest" OR algorithm)
4	Web of Science	TS=("gestational diabetes" OR GDM) AND TS=("type 2 diabetes" OR T2DM) AND TS=("machine learning" OR "artificial intelligence" OR "deep learning" OR "predictive modeling" OR "predictive model" OR "data mining" OR "neural network" OR "support vector machine" OR "random forest" OR algorithm)
